# The Role of the Guanosine Nucleotide-Binding Protein in the Corpus Luteum

**DOI:** 10.3390/ani11061524

**Published:** 2021-05-24

**Authors:** Dody Houston Billhaq, Seunghyung Lee

**Affiliations:** College of Animal Life Sciences, Kangwon National University, Chuncheon 24341, Korea; shung0626@gmail.com

**Keywords:** RAS, ovary, corpus luteum, cows, reproduction

## Abstract

**Simple Summary:**

This review aims to discuss the role of the guanosine nucleotide-binding protein (RAS) family in the biological events that occur during the formation and regression of the corpus luteum in the ovary. RAS proteins mediate extracellular signals, transduce through their receptors via multiple signaling pathways, and regulate a wide array of cellular processes. RAS exhibits a notable function in the regulation of vascular endothelial growth factor, fibroblast growth factor, insulin-like growth factor, angiopoietins (ANPT), and hypoxia-inducible factor (HIF). RAS proteins appear to be involved in several factors that are notably associated with the regulation of the corpus luteum. Further research is necessary to enhance our understanding of the role of the RAS family in the ovarian corpus luteum.

**Abstract:**

The corpus luteum is a temporary endocrine gland in the ovary. In the ovarian cycle, repeated patterns of specific cellular proliferation, differentiation, and transformation occur that accompany the formation and regression of the corpus luteum. Molecular mechanism events in the ovarian microenvironment, such as angiogenesis and apoptosis, are complex. Recently, we focused on the role of RAS protein in the ovarian corpus luteum. RAS protein plays a vital role in the modulation of cell survival, proliferation, and differentiation by molecular pathway signaling. Additionally, reproductive hormones regulate RAS activity in the cellular physiological function of ovarian follicles during pre-ovulatory maturation and ovulation. Thus, we have reviewed the role of RAS protein related to the biological events of the corpus luteum in the ovary.

## 1. Introduction

The ovary is a dynamic female reproductive organ that undergoes many structural and functional transformations as it fulfills its two significant roles of producing female gametes and synthesizing sex steroid hormones [1]. In the ovarian cycle, repeated patterns of specific cellular proliferation, differentiation, and transformation occur that accompany the process of follicular development and the formation and function of the corpus luteum [2]. The ovarian cycle is a highly orchestrated, and complex developmental process that depends on functional interactions and critical signaling pathways that communicate between the ovarian microenvironment elements, including the oocyte, granulosa cells, theca cells, and surrounding stromal components, including vascular and immune cells [3]. In cellular signaling, the monomeric small GTPases (small G proteins) are known to play an important role in diverse molecular processes. The small-G-protein family consists of numerous proteins with varying degree of homology, one of which is a guanosine nucleotide-binding protein (RAS) [4].

RAS family proteins include 23 genes coding for at least 25 proteins that are divided into eight paralog groups based on sequence identity, structure, and function: RAS, RAS-like (RAL), RAS-related protein (R-RAS), RAS-like protein in tissues (RIT), RAS-related protein Rap (RAP), RAS homolog enriched in brain (RHEB), Dexamethasone-induced RAS-related protein (RASD), and GTP-binding protein Di-RAS (DIRAS) [5]. The paralog group of RAS proteins is composed of four isoforms (H-RAS, N-RAS, K-RAS4A, and K-RAS4B) that are encoded by three genes, *H-RAS*, *N-RAS*, and *K-RAS* [6]. RAS family proteins are membrane-associated, small GTPases that have the function of transmitting a multitude of cellular signals [7]. RAS family proteins mediate extracellular signals, transduced through their receptors, with multiple signaling pathways and consequently regulate a wide array of cellular processes [8]. The RAS family proteins act as binary molecular switches that cycle between active guanosine triphosphate (GTP)-bound and inactive guanosine diphosphate (GDP)-bound states [9]. RAS family proteins play a vital role in the modulation of cell survival, proliferation, and differentiation by signaling through a set of molecular pathways [10]. Those molecular functions of RAS family proteins exhibit an essential role that co-occurs in the repeated pattern of physiological change during the ovarian cycle. Moreover, RAS and RAS GTPases are changed in the development and regression of the corpus luteum. However, the function of RAS in the ovarian corpus luteum is not clear. This review aims to discuss the role of RAS family proteins related to biological events in the ovarian corpus luteum. 

## 2. Potential Function of RAS in the Ovary

RAS family proteins function as GDP/GTP regulated on–off switches involved in the regulation of cytoplasmic–nuclear signaling networks controlling diverse normal cellular processes [6]. The molecular “on/off” switch form of RAS family proteins is highly related to the role of guanine nucleotide exchange factors (GEFs) and GTPase-activating proteins (GAPs) that mediate the activation and inactivation form of RAS family proteins [11]. GEFs facilitate RAS conformational change from the inactive GDP-bound form to the active GTP-bound form by promoting nucleotide release [12]. On the other hand, GAPs stimulate the transitional change from the RAS active state into the inactive GDP-bound state by catalyzing RAS-mediated GTP hydrolysis [13]. Those cycling mechanisms between active GTP-bound and inactive GDP-bound are essential molecular switches in cellular signaling events.

In a molecular signaling event, G-protein-coupled receptors (GPCRs) relay various extracellular signals into cells by catalyzing nucleotide release from heterotrimeric G proteins [14]. The small G proteins, RAS, and other GTPases are highly responsive to multiple signal inputs to switch them on and off, allowing the processes of the GEF and GAP cycles [15]. The RAS proteins are governed by a crucial principle—translocation —in that GEFs and GAPs switch RAS on and off while they are recruited to the plasma membrane and positioned in direct proximity to RAS [9]. In its active GTP-bound state, RAS interacts with several effector proteins that initiate downstream signaling during intracellular events [16]. The activation of RAS is mainly related to typical membrane-induced conformational changes promoting RAS functional diversity [17]. In addition, the activation of small G proteins, such as RAS, through plasma membrane receptors is induced by diverse stimuli, including growth factors, antigens, chemokines, cytokine ligands, paracrine agents, and hormones [4]. 

A hormone is a chemical messenger that coordinates the activities of different cells in a multicellular organism [18]. The roles of hormones and other regulatory agents are essential in the regulation of the ovary, in which the hypothalamic–pituitary–gonadal (HPG) axis primarily participates in folliculogenesis, ovulation, and corpus luteum function [19]. The hypothalamus is involved in the neuroendocrine control of ovarian function via secreting a releasing hormone called gonadotropin-releasing hormone (GnRH), produced by the hypothalamus peptidergic neurons [20]. GnRH primarily acts on the anterior pituitary inducing the release of follicle-stimulating hormone (FSH) and luteinizing hormone (LH), which exert their effects on the ovary [21]. The reproductive hormones FSH and LH are critical for many events in the female ovary that regulate follicular development, ovulation, and luteinization [22]. 

FSH and LH are members of the glycoprotein hormone family that share a common α-subunit and differ in their unique β-chains [23]. FSH and LH employ trophic and stimulatory effects on gametogenesis process by binding to a specific receptor located exclusively on the surface membrane of granulosa cells in the ovary; the binding of LH is also important for the synthesis of testosterone in theca cells [24,25]. The receptors of the pituitary gonadotropin hormones FSH and LH, the FSH receptor (FSHR) and LH receptor (LHR, also known as LHCGR), belong to the highly conserved subfamily of the G-protein-coupled receptor (GPCR) superfamily [26]. These receptors harbor a large extracellular domain containing a high-affinity hormone binding site, and a small intracellular domain that is coupled to the signal transduction pathways [27]. The extracellular domain is structurally linked by the hinge region to the transmembrane domain, composed of seven α helices connected by alternating intracellular and extracellular loops and involved in the activation and signaling functionality of the receptors [26]. 

The event of FSH embedding onto the membrane receptor initiates downstream signaling cascades that lead to oocyte maturation in the ovary [28]. Additionally, the activity of FSH is dependent on glycosylation in the gonads and is stimulated by FSHR activation via the Gα_s_/cAMP/PKA signaling pathway. In cellular signaling, the disruption or modification of FSH signaling through mutation and polymorphism of FSHR, causing subfertility or infertility, has been denoted [29]. FSHR is involved in several primary signal transduction mechanisms, including the activation of protein kinase A (PKA); however, PKB and PKC are also involved [25]. The broad range of PKA-dependent signaling pathways indicates that kinase is the primary regulator of several FSH-dependent cell functions that are highly associated with steroidogenesis and cell differentiation [30]. The FSH stimulus transduces the intracellular signaling through the FSHR-mediated pathway, which leads to an increase in intracellular second messenger molecules (cAMP) and the activation of PKA [26]. In a recent investigation of the FSH receptor-binding inhibitor, K-RAS was shown to play an essential role in regulating FSH-induced cAMP production and PKA expression in ovarian granulosa cells [31,32]. Furthermore, PKA mediates the actions of FSH on granulosa cell membranes by signaling through multiple targets to activate the PI3K/AKT pathway, which leads to increased proliferation, inhibition of apoptosis, enhanced translation, and activation of target genes with products involved in the maturation process of pre-ovulatory granulosa cells [33].

**Figure 1:**
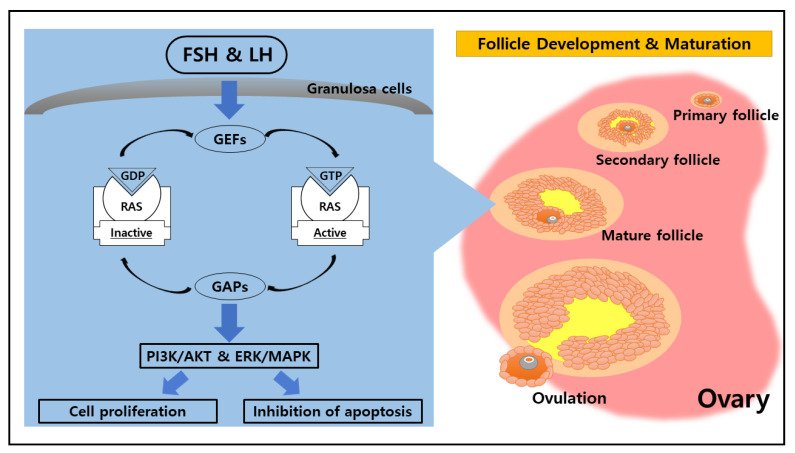
Role of RAS in the ovarian physiological cycle. Ovarian function is regulated by chemical messengers such as follicle-stimulating hormone (FSH) and luteinizing hormone (LH). Both FSH and LH coordinate cellular activities through the small G protein (RAS), which mediates the upstream signal from FSH and LH to regulate the PI3K/AKT and ERK/MAPK pathways. The activation of those pathways leads to cell proliferation and inhibition of apoptosis, which is essential for follicle development and maturation as well as ovulation within the ovary.

In parallel to the FSH role, LH simultaneously participates in the ovarian follicular maturation process through induction, which is followed by ovulation [34]. The ovarian follicular responses to LH require the participation of oocyte-derived paracrine factors and are mediated by epidermal growth factor (EGF)-like growth factors, including amphiregulin (Areg) and epiregulin (Ereg), produced by granulosa cells [35]. LH-mediated GPCR signaling on granulosa cells results in the upregulation of specific EGF ligands in the activation of the Erk signaling cascade that occurs downstream of cAMP [36]. The LH influence in granulosa cells initiates the LH-regulated signaling pathway, which promotes the expression of EGF-like factors (Areg and Ereg) through the activation of EGF receptors (EGFR), RAS, and extracellular signal-regulated kinase 1 and 2 (ERK 1/2, also known as mitogen-activated protein kinase 3 and 1 (MAPK3/1)) [37]. In response to hormone-induced ovulation, the activated RAS was significantly increased in transient pattern expression after 2 h of treatment of human chorionic gonadotropin (hCG), the LH analog, which induces ovulation [38]. Both LH and hCG are heterodimeric glycoprotein hormones that bind to the same receptor [39]. Furthermore, RAS, notably K-RAS, is an essential intermediary of gonadotropin signaling in ovarian follicles [40]. The signal transduction that acts upon the reproductive hormones illustrates that RAS is basically involved in all the cellular physiological functions of ovarian follicles during pre-ovulatory maturation and ovulation within the ovary (Figure 1). However, the role of RAS-regulating LH is not clear in theca cells, so we need to research its function in this cell type.

## 3. The Likelihood of a Novel Role of RAS in the Ovarian Corpus Luteum

The ovarian corpus luteum is a transient endocrine gland with the primary function of producing and secreting the progesterone required for pregnancy [41]. This remarkable transient endocrine tissue is derived from the ovulated follicle [42]. Subsequent to ovulation and in response to the LH surge, the ovarian follicle undergoes luteinization, which involves the modulation of gene expression, vast cellular proliferation, and differentiation of granulosa and theca cells, transforming these cells into the large and small luteal steroidogenic cells [43]. The transformation of granulosa and theca cells into luteal cells is accompanied by hypertrophy and hyperplasia of the cells, which leads to an increase in cell size and the number of luteal cells [44]. The switch from a pre-ovulatory follicle to a fully functional ovarian corpus luteum is a notable and dynamic biological process that requires a strictly coordinated series of events following ovulation [45]. In the course of its lifespan, the corpus luteum undergoes three major stages of physiological alteration, including the formation, function, and regression that are principal features of pregnancy and the reproductive cycle [46]. 

The continuation of development from ovulatory follicle to corpus luteum is characterized by remarkable growth, differentiation, and remodeling [47]. Throughout this process, the development of new microvasculature is required for luteal structure and function [48]. The vascular system develops via two disparate mechanisms known as vasculogenesis and angiogenesis, in which the mechanism of angiogenesis is required for the corpus luteum blood vessel growth [49]. In the early and mid-luteal phase of the corpus luteum life cycle, the ovarian corpus luteum undergoes a high level of neovascularization in major sites of angiogenesis [42]. Angiogenesis is defined as the formation of new capillaries from pre-existing blood vessels by migration, proliferation, and three-dimensional arrangement of endothelial cells [50]. In the process of luteal angiogenesis, the follicle rupture initiates the formation of new capillaries through the breakdown of the basement membrane that enables blood vessels to invade the granulosa layers as cellular remodeling begins [51]. This vascularization process during luteal angiogenesis is critical for the delivery of adequate levels of hormones and other nourishing substances required to support the corpus luteum function [48]. The angiogenesis mechanism is highly regulated under several factors, involving the orchestrated balance between pro- and anti-angiogenic elements [52]. 

The establishment of new capillaries from a preexisting vascular network is governed by a variety of factors, including angiogenic factors that act directly on vascular endothelial cells to stimulate locomotion and act indirectly by immobilizing host cells to release endothelial growth factors [53,54]. Of the numerous angiogenic factors that have been identified, the dominant factors associated with angiogenesis in ovarian follicles and corpus luteum appear to be vascular endothelial growth factor (VEGF), insulin-like growth factor (IGF), fibroblast growth factor (FGF), angiopoietins (ANPT), and hypoxia-inducible factor (HIF) family member [2]. In the angiogenesis mechanism, VEGF is the most potent angiogenic factor that acts as a critical regulator of physiological angiogenesis during embryogenesis, skeletal growth, and reproductive function [55]. VEGFs are abundantly expressed, structurally related dimeric molecules that play crucial roles in the formation, function, and maintenance of the vasculature in ovarian corpus luteum [56,57]. In addition, the IGF system may have direct or indirect effects on angiogenesis by inducing actions for VEGF production in luteal cells as well as by proliferation and differentiation of endothelial cells [58]. During the luteal development, FGF, which belongs to the heparin-binding factor family, also plays a crucial factor in the angiogenesis and neovascularization of ovarian corpus luteum [59]. In the corpus luteum vessel stabilization and maturation process, ANPT plays a role in the interaction between endothelial cells and the surrounding matrix, which is crucial to the regulation of the integrity of the microvascular network [60]. Furthermore, HIF primarily contributes to ovarian angiogenesis due to its interaction and response to the hypoxic microenvironment of the corpus luteum. HIF plays a vital role in the angiogenesis of rapid-growing tissue via transcriptional regulation of angiogenic factors such as VEGF (Table 1) [61].

In previous research on corpus luteum formation, VEGF was found to be strongly expressed in both small and large luteal cells during the early and middle luteal phase, characterized by an intense proliferation index and dense network of capillaries [62,63]. Similar expression patterns of angiogenic promoters, IGF and FGF, were discovered to be increased during the early and middle stages of the luteal phase [64]. In addition, HIF was highly expressed during the early stages of the luteal phase, and HIF notably contributes to mediating and regulating VEGF expression, which involves VEGF-dependent luteal development [65,66]. Compared to three other angiogenic factors that have already been mentioned, ANPT exhibits nonsimilar patterns. The expression of ANPT was upregulated during the middle and late luteal phase, which indicates that ANPT is necessary to maintain and stabilize the blood vessels [67]. However, those essential proteins are involved not only in corpus luteum formation but also in the regulation of corpus luteum regression, reported in certain previous studies.

At the end of the lifespan of the corpus luteum, a regression process will occur that leads to this transient endocrine organ’s disappearance from the ovary [68]. The corpus luteum regression, termed luteolysis, begins when the oocyte is not fertilized; this is its major signal to undergo degeneration, allowing the initiation of a new ovarian cycle [69]. The main factor that induces the luteolysis of the ovarian corpus luteum is prostaglandin F2α hormone (PGF2α), secreted by the uterus, which reaches the corpus luteum via a countercurrent system between the uterine vein and the ovarian artery [70]. The corpus luteum regression consists of two primary elements: functional luteolysis, indicated by the reduction of progesterone hormone production; and structural luteolysis, marked by tissue degeneration and cell death [71]. Subsequent to the onset of functional luteolysis, structural luteolysis occurs, promoted by the severe and chronic termination of the blood supply, which leads to cell death or apoptosis in both luteal and endothelial cells under the direct and/or indirect action of vasoactive substances [72].

Vasoactive substances such as the FGF and VEGF reported in former research on corpus luteum regression were found to have a remarkable effect on expression patterns in that FGF was significantly upregulated during functional luteolysis and involved in reducing the inflammatory response and counterregulating the mechanism of corpus luteum regression; however, FGF was notably downregulated during structural luteolysis, along with VEGF [73]. VEGF expression, mediated by HIF, is a vital mechanism in ovarian corpus luteum regulation, and both of the VEGF and HIF expressions were significantly reduced during the period of corpus luteum luteolysis [74]. In addition, changes in mRNA expression of VEGF in luteal vascular cells were positively correlated with alterations in ANPT, indicating a functional relationship that mainly participates in regulating the tissue remodeling of the vascular bed in order to aid luteal regression [75]. Another vasoactive substance like IGF exhibited similar expression patterns and appeared to have an identical role to FGF during the corpus luteum regression, in which the IGF was upregulated during the functional luteolysis; meanwhile, when the structural luteolysis began, IGF expression was significantly reduced, which indicated the termination of supporting the functional corpus luteum [76]. Furthermore, drastic changes in vasoactive key factors indicate that the modulation of vascular stability is a critical component in the cascade of events that lead to functional and structural luteolysis in ovarian corpus luteum regulation [77]. 

In cellular regulation, the RAS family of GTPases (H-RAS (Ha-RAS), N-RAS (neuroblastoma-RAS), and K-RAS (Ki-RAS)) are known to have an essential role in cellular signal transduction [78]. RAS family proteins transduce signals that control diverse cell functions through distinct effectors, including RAS-activated factor (serine/threonine kinase), phosphatidylinositol 3-kinase (PI3K), RAL-guanine nucleotide dissociation stimulator (GDS), and RAS-guanine nucleotide release protein (GAP) [40]. In tumorigenesis, the RAS family proteins appear to have a close association with neoplasia development of a tumor [82]. Remarkable growth, differentiation, and remodeling occurred in fast-growing tumors compared to ovarian corpus luteum formation, in which the dramatic growth of both tumor and corpus luteum are reliant on a similar mechanism, angiogenesis, the formation of new blood vessels from preexisting vessels mechanism [47]. 

RAS family proteins play a vital role in tissue vasculature establishment through inducing a number of effector pathways that culminate in the transcriptional activation of genes that regulate cellular angiogenesis [83]. That RAS family proteins involved in the regulation of the proangiogenic factor, i.e., the mutants H-RAS and K-RAS, have been correlated with increased VEGF expression demonstrates that the induction of VEGF by hypoxia is modulated by a PI3K-AKT pathway in H-RAS-transformed cells via a HIF-1 transcriptional element [84]. RAS was profoundly involved in the regulation of HIF expression, not only inducing the upregulation of VEGF but also facilitating cell migration and invasion [85]. In the regulation of vascular morphogenesis and physiology, ANPT plays a pivotal role in the complex process of blood vessel maturation, acting cooperatively with VEGF and having a dependent association with RAS family activation [86]. Moreover, a small GTPase of the RAS family proteins, R-RAS, is a vital regulator of vessel integrity and function during tumor vascularization, in which R-RAS promotes endothelial barrier function and pericyte association with nascent blood vessels by blocking plasma leakage and improving blood perfusion and the blood vessel structure [87]. 

**Figure 2:**
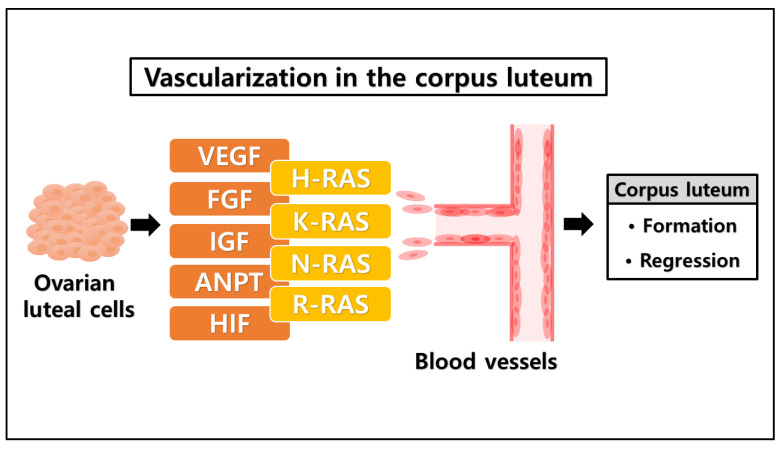
The possible link between RAS family and angiogenic factors in ovarian corpus luteum regulation. Diverse factors are involved in the ovarian corpus luteum regulation. VEGF, FGF, IGF, ANPT, and HIF are dominant factors expressed by the luteal cells and play an important role in the process of blood vessel vascularization via angiogenesis, required for the corpus luteum formation and regression. Those angiogenic factors have a significant correlation with the RAS family (H-RAS, K-RAS, N-RAS, and R-RAS), in which the RAS family mainly participates in tissue vasculature development. Taken together, the RAS family may be involved in the process of luteal angiogenesis in the ovarian corpus luteum.

Correspondingly, the RAS family proteins are essential elements in multiple growth factor signaling pathways. In a previous investigation of IGF signal transduction, K-RAS activation as a downstream signal was disrupted by the blockade of the IGF receptor, which terminated the IGF signal for inducing cell proliferation [88]. Furthermore, the knockdown of K-RAS effectively abolished the upstream signal of IGF, disabling IGF’s ability to trigger the activation of the ERK pathway [79]. In a variety of cellular processes like cell migration, cell proliferation, and angiogenesis, FGF signals are transduced to the main signaling cascade, including the RAS-MAPK and PI3K-AKT signaling pathway [80]. Former research into FGF signaling reported that the inhibition of FGF activity leads to the inactivation of RAS-MAPK and PI3K-AKT, blocking the cellular FGF activity in the cell migration process [81]. Furthermore, our recent investigation discovered that the expression of H-RAS and R-RAS was upregulated during the early phase of corpus luteum formation, which indicates that RAS family proteins may be required for the proliferation and differentiation of luteal cells [89]. The RAS family proteins appear to be involved in several factors that are notably associated with ovarian corpus luteum regulation (Figure 2).

## 4. Conclusions

The regulation of angiogenic factors and the RAS family is important during follicular growth, ovulation, and the formation and regression of the corpus luteum. A number of factors, including chemical messengers and ligands, are involved in ovarian function. Among the factors involved in ovarian function, angiogenic factors such as VEGF, FGF, IGF, ANPT, and HIF are dominant factors that play a pivotal role in the orchestration of the blood vascularization process throughout corpus luteum formation and regression. Furthermore, the RAS family has a notable function in the regulation of VEGF, FGF, IGF, ANPT, and HIF. The approaches elucidated above indicate a strong association between the RAS family and those dominant factors, which points to the involvement of the RAS family in the regulation of the ovarian corpus luteum. Nevertheless, further research is necessary to enhance our understanding of the RAS family’s role in the ovarian corpus luteum. Moreover, RAS may be key to identifying complex tracking mechanisms in the biological function of the corpus luteum.

## Figures and Tables

**Table 1:** Various factors and potential factors in the corpus luteum.

Conditions	Factors	Functions
Formation of corpus luteum	Vascular endothelial growth factor (VEGF)	Vasculogenesis [57]
	Insulin-like growth factor (IGF)	Angiogenesis [58]
	Fibroblast growth factor (FGF)	Angiogenic factor [59]
	Angiopoietins (ANPT)	Cellular growth factor [60]
	Hypoxia-inducible factor (HIF)	Transcription [61]
Regression of corpus luteum	Prostaglandin F2α (PGF2α)	Luteolysis [70] Apoptosis [71]
RAS family	H-RAS	Cellular signal transduction [78]
	K-RAS	ERK pathway [79]
	N-RAS	PI3K-AKT pathway [80]
	R-RAS	RAS-MAPK pathway [81]

## Data Availability

No new data were created or analyzed in this study. Data sharing is not applicable to this article.
